# Savages’ Notions of Nightmare

**Published:** 1882-03

**Authors:** 


### SAVAGES' NOTIONS OF NIGHTMARE.

One, however, of the most popular notions of nightmare is that it is a demon or fiend, who takes advantage of the hours of darkness, when special license is supposed to be given to beings of the ghostly world to take their walks abroad, to ride through benighted districts, in order either to throttle some snoring peasant, or to make itself actually present in his dreams, and thereby intensify their grim reality. Indeed, this idea is found even among savage tribes, and may be traced back to a very remote period. Thus, by way of example, we are told how the North American Indians, after a night of debauchery and excessive feasting, are said to be visited by nocturnal visitors of a not very agreeable kind, who, with threatening gestures, scare their sleeping victims. The Caribs, when subjected to hideous dreams, often on awakening have declared that the demon Maboya has ill-treated and beaten them in their sleep, affirming that they could even still feel the effects of his rough treatment. Hence, in the early days of Christianty, the idea found favor in many eyes that the demon of nightmare was one of the means which Satan employed for molesting and seducing human souls. Persons, therefore, whose slumbers had been broken by impure and unholy dreams were believed to have been unconsciously under the influence of Satan's sway, and to have indulged in sinful desires and inclinations.

Among the many tales which illustrate the theory of the nightmare as being a demon, we may briefly relate a Netherlandish one which is a fair example of others of a similar kind. Two young men were in love with the same lady. One of them being tormented every night by a nightmare, sought advice from his rival, who took advantage of his act of confidence, and gave him the subjoined piece of treacherous advice: "Hold a sharp knife with the point toward your breast, and you'll never see the Mara again." His comrade thanked him, but on retiring to rest he thought it as well to be on the safe side, and so held the knife handle downward. Consequently, when at midnight the Mara made her accustomed visit, instead of forcing the knife into his breast, she cut herself badly, and escaped from the room making a terrible noise. The legend unfortunately does not tell us the issue of this tragic affair, but we can only hope that the young man revenged himself on his false and malicious rival by promptly marrying the young lady.—*Gentleman's Magazine.*

How to Treat a Cold.—When you get chilly all over and away into your bones, and begin to sniffle and almost struggle for your breath, just begin in time and your tribulation need not last very long. Get some powdered borax and snuff the dry powder up your nostrils. Get your camphor bottle and smell it frequently; pour some on your handkerchief, and wipe your nose with it whenever needed. Your nose will not get sore, and you will soon wonder what has become of your cold. Begin this treatment in the forenoon and keep on at intervals until you go to bed, and you will sleep as well as you ever did.

### BOYS.

GET hold of the boy's heart. Yonder locomotive comes like a whirlwind down the track, and a regiment of armed men might seek to arrest it in vain. It would crush them, and plunge unheeding on. But there is a little lever in its mechanism that at the pressure of a man's hand will slacken its speed, and in a moment or two bring it panting and still, like a whipped spaniel, at your feet. By the same little lever the vast steamship is guided hither and yon, upon the sea, in spite of adverse wind or current. That sensitive and responsive spot by which a boy's life is controlled is his heart. With your grasp gently and firm on that helm, you may pilot him whither you will. Never doubt that he has a heart. Bad and willful boys very often have the tenderest heart hidden away somewhere beneath incrustations of sin or behind barricades of pride. And it is your business to get at that heart, keep hold of it by sympathy, confiding in him, manifestly working only for his good by little indirect kindnesses to his mother or sister, or even his pet dog. See him at his home, or invite him into yours. Provide him some little pleasure, set him at some little service of trust for you; love him; love practically. Any way and every way rule him through the heart.

